# Transboundary Movement of Yezo Virus via Ticks on Migratory Birds, Japan, 2020–2021

**DOI:** 10.3201/eid3012.240539

**Published:** 2024-12

**Authors:** Ayano Nishino, Kango Tatemoto, Keita Ishijima, Yusuke Inoue, Eun-sil Park, Tsukasa Yamamoto, Masakatsu Taira, Yudai Kuroda, Milagros Virhuez-Mendoza, Michiko Harada, Noboru Nakamura, Gen Morimoto, Hiroki Yamaguchi, Takuma Ariizumi, Ai Takano, Hiroshi Shimoda, Keita Matsuno, Ken Maeda

**Affiliations:** National Institute of Infectious Diseases, Tokyo, Japan (A. Nishino, K. Tatemoto, K. Ishijima, Y. Inoue, E. Park, T. Yamamoto, M. Taira, Y. Kuroda, M. Virhuez-Mendoza, M. Harada, K. Maeda); Yamaguchi University, Yamaguchi, Japan (A. Nishino, T. Yamamoto, M. Harada, A. Takano, H. Shimoda, K. Maeda); Yamashina Institute for Ornithology, Chiba, Japan (N. Nakamura, G. Morimoto); Hokkaido Institute of Public Health, Hokkaido, Japan (H. Yamaguchi); Hokkaido University, Hokkaido (T. Ariizumi, K. Matsuno)

**Keywords:** Yezo virus, ticks, wild birds, tickborne diseases, vector-borne infections, viruses, zoonoses, Japan, *Ixodes persulcatus*, *Emberiza spodocephala*

## Abstract

Migratory birds carry ticks harboring various pathogens, including the zoonotic Yezo virus. In Hokkaido, Japan, we collected ticks from migratory birds during 2020–2021. Eight of 385 pools, comprising 2,534 ticks, tested positive for Yezo virus RNA, suggesting Yezo virus might be spread through the flyways of migratory birds.

Yezo virus (YEZV), in the order Bunyavirales, family Nairoviridae, genus *Orthonairovirus*, possesses a negative-sense single-stranded RNA genome comprising 3 segments: large (L), medium (M), and small (S) (*1*). Each segment contains a single open reading frame encoding the RNA-dependent RNA polymerase (L segment), glycoprotein precursor (M segment), and nucleoprotein (S segment) (*1*). YEZV infection is an emerging infectious disease, detected in Hokkaido, Japan, in 2019 among patients who had febrile illness after a tick bite (*1*); >9 patients infected with YEZV have been reported in Hokkaido (*1*–*3*). Among those patients, fever, myalgia, thrombocytopenia, leukopenia, and increasing liver enzymes were commonly reported after a tick bite (*1*–*3*). One patient who had a YEZV infection after a tick bite has also reported in Inner Mongolia in northeastern China (*4*).

Migratory birds carry ticks harboring various pathogens, such as Crimean-Congo hemorrhagic fever virus, belonging to the family *Nairoviridae* (*5*–*7*). Phylogenetic analysis of tickborne severe fever with thrombocytopenia syndrome virus indicated that this virus might be carried by ticks found on migratory birds that fly between China, South Korea, and Japan (*8*,*9*). Increasing evidence suggests that migratory birds play a critical role in spreading tickborne pathogens. In archipelagos, such as Japan, understanding transmission of pathogens by migratory birds is critical; however, information on tickborne pathogens carried by migratory birds is lacking. We investigated the prevalence of YEZV in ticks found on migratory birds in Japan to determine virus transmission pathways.

## The Study

**Figure 1:**
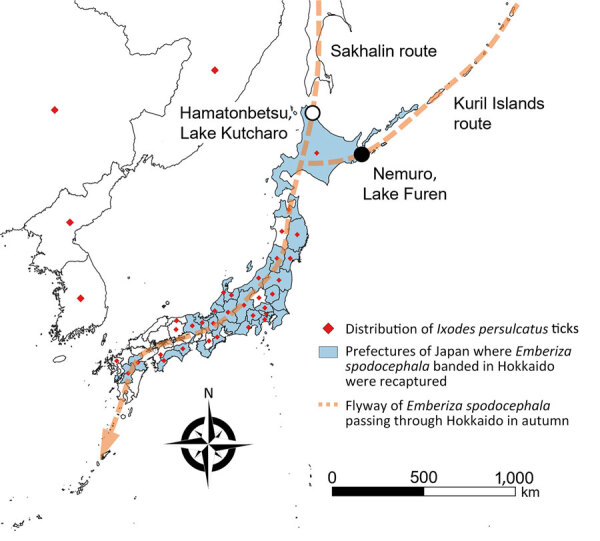
Migratory bird flyways and distribution of *Ixodes persulcatus* ticks in study of transboundary movement of Yezo virus via ticks on birds, Japan, 2020–2021. Distribution of *I. persulcatus* ticks (red rhombus), prefectures of Japan where black-faced buntings (*Emberiza spodocephala*) banded in Hokkaido were recaptured (light blue), and flyway of *E. spodocephala* passing through Hokkaido during autumn (orange dotted line). Distribution outside Japan is indicated at the country level. Black circle shows Lake Furen, Nemuro City, Hokkaido; white circle shows Lake Kutcharo, Hamatonbetsu Town, Hokkaido.

We conducted this research under approval by the animal research review board of Yamashina Institute for Ornithology, Chiba, Japan (approval nos. 2020-004 and 2021-002). We collected ticks infesting birds that mostly fly from Sakhalin and the Kuril Islands to Hokkaido, Japan. We collected the ticks during October 2–12, 2020, and October 2–13, 2021, in Lake Kutcharo, Hamatonbetsu Town, and Lake Furen, Nemuro City, Japan (Figure 1). We analyzed YEZV genes in the ticks to clarify virus spread. Because migratory birds at these locations in autumn are considered to have just arrived from Sakhalin (Sakhalin route) and the Kuril Islands (Kuril Islands route) (Figure 1), it is likely that the ticks crossed the sea attached to the birds. 

We collected 2,534 ticks from 15 species of birds in October 2020 and 2021 (Appendix Tables 1, 2). All ticks were morphologically identified and pooled according to their species, life stage, and host species. Each pool consisted of <10 larval or 5 nymphal ticks (Appendix).

**Table 1:** Virus prevalence in tick species collected in an investigation of transboundary movement of Yezo virus via ticks on migratory birds, Japan, 2020–2021

Species	No. ticks examined	No. pools examined	No. positive pools	Minimum infection rate, %
*Ixodes persulcatus*				
Larva	1,727	193	3	0.2
Nymph	596	137	5	0.8
*I*. *pavlovskyi*				
Larva	169	30	0	0
Nymph	34	19	0	0
*I*. *turdus*				
Larva	0	0	0	0
Nymph	1	1	0	0
*Haemaphysalis megaspinosa*				
Larva	3	2	0	0
Nymph	0	0	0	0
*H*. *concinna*				
Larva	3	2	0	0
Nymph	1	1	0	0
Total	2,534	385	8	0.3

**Table 2:** Genomic sequences in an investigation of transboundary movement of Yezo virus via ticks on migratory birds, Japan, 2020–2021*

Virus strain	Tick stage	No. ticks in pool	Collection site	Collection date	Segment	Accession nos.†
YEZV/tick/BT-1821/Japan/2020	Nymph	5	Lake Kutcharo, Hamatonbetsu	2020 Oct 6–7	L	LC735725
					M	LC735726
					S	LC735727
YEZV/tick/BT-1822/Japan/2020	Nymph	5	Lake Kutcharo, Hamatonbetsu	2020 Oct 6–7	S	LC737964, partial sequence
YEZV/tick/BT-1826/Japan/2020	Nymph	5	Lake Kutcharo, Hamatonbetsu	2020 Oct 7	L	LC735728
					M	LC735729
					S	LC735730
YEZV/tick/BT-1844/Japan/2020	Larva	10	Lake Furen, Nemuro	2020 Oct 8	L	LC735731
					M	LC735732
					S	LC735733
YEZV/tick/BT-1864/Japan/2020	Larva	10	Lake Furen, Nemuro	2020 Oct 8	L	LC735734
					M	LC735735
					S	LC735736
YEZV/tick/BT-1968/Japan/2020	Larva	10	Lake Furen, Nemuro	2020 Oct 11	L	LC790674
					M	LC790675
					S	LC790676
YEZV/tick/BT-2135/Japan/2021	Nymph	5	Lake Furen, Nemuro	2021 Oct 13	L	LC790677
					M	LC790678
					S	LC790679
YEZV/tick/BT-2155/Japan/2021	Nymph	5	Lake Furen, Nemuro	2021 Oct 13	L	LC790680
					M	LC790681
					S	LC790682

*All ticks collected were *Ixodes persulcatus* and all birds collected were black-faced buntings (*Emberiza spodocephala*). L, large segment; M, medium segment; S, small segment. †DNA Data Bank of Japan (https://www.ddbj.nig.ac.jp) accession numbers.

We examined 2,323 *Ixodes persulcatus* ticks (1,727 larvae and 596 nymphs), 203 *I*. *pavlovskyi* ticks (169 larvae and 34 nymphs), 1 *I*. *turdus* tick (nymph), 3 *Haemaphysalis megaspinosa* ticks (larvae), and 4 *H*. *concinna* ticks (3 larvae and 1 nymph) by using quantitative reverse transcription PCR (Appendix). Of those, 8 pools of *I. persulcatus* ticks were positive for YEZV RNA; no pools for the other tick species were YEZV positive (overall minimum infection rate 0.3%) (Table 1). Seven of 8 positive pools showed low cycle threshold (Ct) values of 22.08–30.08; 1 pool had a high Ct value of 39.19. Seven pools with low Ct values were used for further analysis. Ticks in all positive pools were *I. persulcatus* collected from black-faced buntings (*Emberiza spodocephala*) (Table 2). Five pools were collected in Lake Furen, Nemuro City, Hokkaido, and the other pools were collected in Lake Kutcharo, Hamatonbetsu Town, Hokkaido. Results indicated that ticks with YEZV were transferred by migratory birds along both the Sakhalin and Kuril Islands routes.

In *I. persulcatus* ticks, the minimum infection rate of YEZV was 0.2% in larvae and 0.8% in nymphs (Table 1). YEZV has been reported in 3 adult tick species, *H. megaspinosa*, *I. ovatus*, and *I. persulcatus*, found on vegetation in Hokkaido (*1*). In addition, 4 YEZV–positive pools consisted of only unfed ticks (Appendix Table 3), indicating that *I. persulcatus* might transmit Yezo virus.

We sequenced 7 complete genomes of YEZV: YEZV/tick/BT-1821/Japan/2020, YEZV/tick/BT-1826/Japan/2020, YEZV/tick/BT-1844/Japan/2020, YEZV/tick/BT-1864/Japan/2020, YEZV/tick/BT-1968/Japan/2020, YEZV/tick/BT-2135/Japan/2021, and YEZV/tick/BT-2155/Japan/2021 (Appendix) and deposited those sequences into the DNA Data Bank of Japan (https://www.ddbj.nig.ac.jp) (Table 2). For 1 pool that had a high Ct value, YEZV/tick/BT-1822/Japan/2020, we could not determine the complete genome sequence; however, we amplified a short fragment of the S segment by nested reverse transcription PCR and deposited that sequence in the DNA Data Bank of Japan (Table 2).

We analyzed the nucleotide and amino acid sequences of the RNA-dependent RNA polymerase, glycoprotein precursor, and nucleoprotein among YEZV strains and compared those sequences to Sulina virus IxriSL16–01 sequences (*10*) (Appendix Tables 4–6). Sequence identity between YEZV strains was 93.0%–100.0% at the nucleotide level and 99.2%–100.0% at the amino acid level.

**Figure 2:**
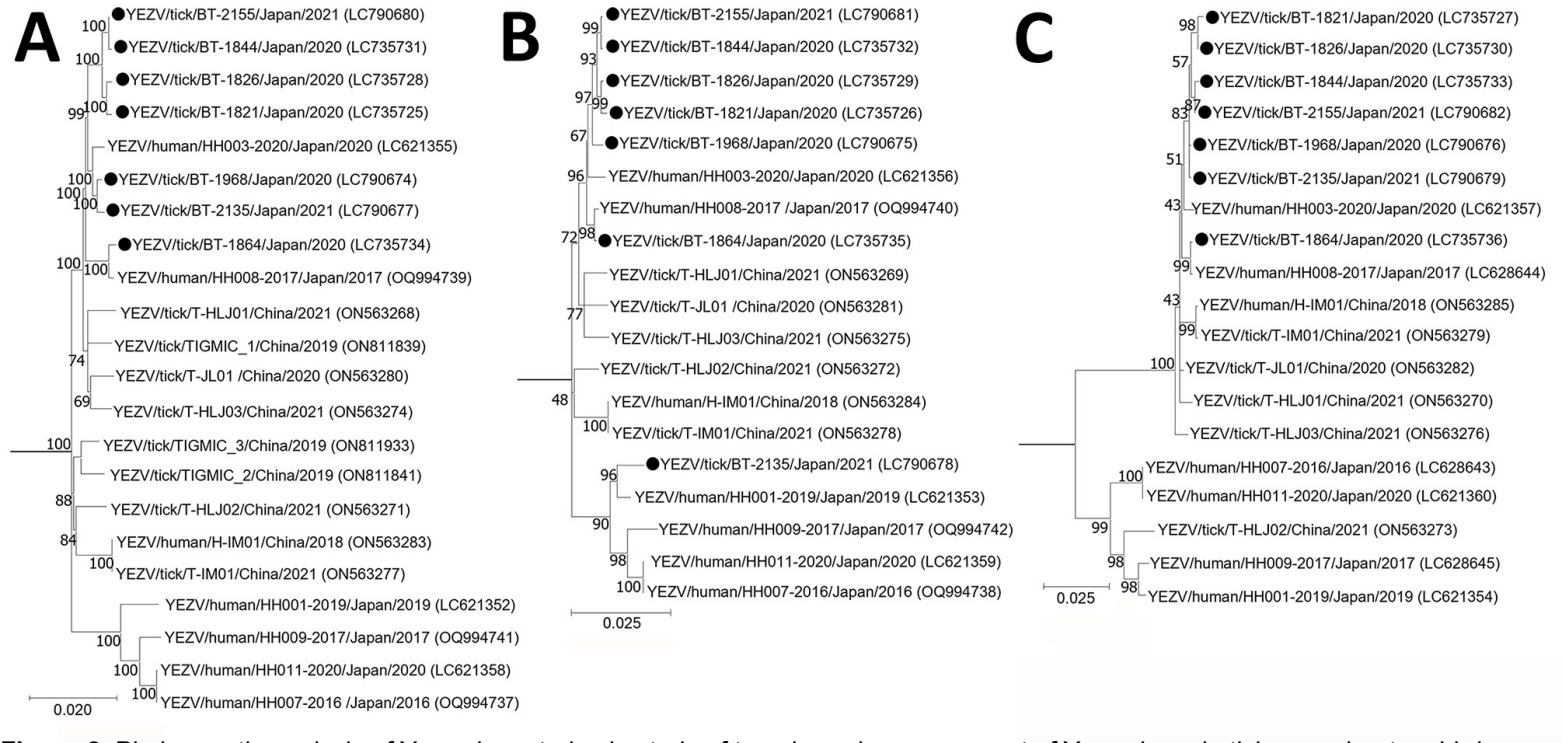
Phylogenetic analysis of Yezo virus strains in study of transboundary movement of Yezo virus via ticks on migratory birds, Japan, 2020–2021. Trees were constructed by using the maximum-likelihood method in MEGA X (https://www.megasoftware.net) and 1,000 bootstrap replicates for nucleotide sequences. A) Large segment; B) medium segment; C) small segment. Black circles indicate sequences from this study. The number at each branch indicates the bootstrap value. GenBank accession numbers for nucleotide sequences are shown in parentheses. Sulina virus IxriSL16-01 was used as the outgroup to determine the root of Yezo virus trees but is not shown. Scale bars indicate nucleotide substitutions per site.

We also performed phylogenetic analysis of YEZV strains by using the nucleotide sequences of the open reading frames of the L, M, and S segments (Figure 2). All tick-derived strains detected in this study belonged to the same cluster as the strains obtained from patients in Hokkaido, YEZV/human/HH003-2020/Japan/2020 and YEZV/human/HH008-2017/Japan/2017 (*1*). In addition, our tick-derived strain, YEZV/tick/BT-2135/Japan/2021, and the tick-derived strain from Heilongjiang, China, YEZV/tick/T-HLJ02/People’s Republic of China/2021, were reassortment strains; both were generated through reassortment with the strains found in patients in Hokkaido, Japan. We also confirmed the events of genetic reassortment by using a recombination detection program (A. Nishino and K. Maeda, unpub. data), which suggested that YEZV might be transferred between China and Hokkaido, Japan.

## Conclusions

We successfully detected YEZV in *I. persulcatus* ticks from migratory birds (*E. spodocephala* buntings) flying to Hokkaido, Japan, from Sakhalin and the Kuril Islands (*5*,*11*). The distribution of *I. persulcatus* ticks partially overlapped with the flyway of *E. spodocephala* buntings passing through Hokkaido in autumn (*12**-**15*). Together with the possible genetic reassortment event between YEZV strains from Japan and China, this finding indicated that YEZV might be carried by migratory birds from other countries to Japan and from Hokkaido to other prefectures in Japan. To elucidate the distribution area and transmission routes of YEZV, further surveillance of YEZV infection should be conducted in ticks, birds, and wild and domestic animals.

Appendix Additional information for transboundary movement of Yezo virus via ticks on migratory birds, Japan, 2020–2021.
