# Supplementary material for: Transboundary Movement of Yezo Virus via Ticks on Migratory Birds, Japan, 2020–2021
Source: Emerg Infect Dis. 2024 Dec;30(12):2674–8. doi: 10.3201/eid3012.240539 (PMC11616666; doi:10.3201/eid3012.240539)
Supplement: Appendix — Additional information for transboundary movement of Yezo virus via ticks on migratory birds, Japan, 2020–2021. [file 24-0539-Techapp-s1.pdf]

DOI: https://10.3201/eid3012.240539

EID cannot ensure accessibility for supplementary materials supplied by authors. Readers who have difficulty accessing supplementary content should contact the authors for assistance.

Transbounday Movement of Yezo Virus via Ticks on Migratory Birds, Japan, 2020–2021

Appendix

Additional Methods

Homogenization of Ticks

The pooled ticks were homogenized in 500 µL of phosphate-buffered saline by using a BioMasher II (Nippi. Inc., https://www.nippi-inc.co.jp). The homogenates were centrifuged at 6,300 × g at 4°C for 10 minutes, and supernatants were filtrated and stored at −80°C until use.

RNA Extraction and Quantitative Reverse Transcription PCR (qRT-PCR)

RNA was extracted from each supernatant by using the MagMAX Viral/Pathogen Nucleic Acid Isolation Kit (Thermo Fisher Scientific, https://www.thermofisher.com). Yezo virus S segment RNAs were amplified by using the QuantiTect Probe RT-PCR Kit (primer 1: 5′-AGCCCTTGACACTGCATTT-3′, primer 2: 5′-CATACAGGAAGGCCATCTCATT-3′, and probe: 6-FAM-ACCTACTACTGGATGTGGAAGGCAGA-3IABkFQ) (QIAGEN, https://www.qiagen.com) under the following thermal conditions: 50°C for 30 minutes, 95°C for 15 minutes, and 45 cycles of 94°C for 15 seconds and 60°C for 60 seconds.

Determination of Yezo Virus Complete Genome Sequences

To determine the complete genome sequences of Yezo viruses (YEZVs), we performed qRT-PCR by using a series of primer sets designed according to deposited nucleotide sequences of YEZV strains. RT-PCR was performed by first using the One Step RT-PCR Kit (QIAGEN), or cDNA was synthesized from RNA by using SuperScript III First-Strand Synthesis SuperMix (Thermo Fisher Scientific); PCR was performed by using Tks Gflex DNA Polymerase (Takara Bio, https://www.takarabio.com). Sanger sequencing was performed by Eurofins Genomics, Japan, (https://www.eurofins.com) on purified PCR products from 4 viruses. The other 3 viruses were sequenced by using next-generation sequencing, and libraries of the purified PCR products were constructed by using the QIAseq FX DNA Library Kit (QIAGEN). The libraries were pooled and purified by using Agencourt AMPure XP (Beckman Coulter, Inc., https://www.beckman.com). Sequencing was performed by using MiSeq and MiSeq Reagent Nano Kit v2 (both Illumina, https://www.illumina.com). All reads were mapped to the YEZV HH003-2020 strain and consensus sequences were obtained by using CLC Genomics Workbench software (QIAGEN).

Appendix Table 1. Information on migratory birds captured in Japan, 2020, and the infesting ticks

Species of migratory bird	No. captured birds			No. birds infested by ticks*			No. ticks*		
	Hamatonbetsu (Lake Kutcharo)	Nemuro (Lake Furen)	Total	Hamatonbetsu (Lake Kutcharo)	Nemuro (Lake Furen)	Total	Hamatonbetsu (Lake Kutcharo)	Nemuro (Lake Furen)	Total
Emberiza spodocephala	693	1,142	1,835	129	519	648	185	991	1,176
Horornis diphone	100	36	136	15	2	17	16	2	18
Turdus chrysolaus	4	35	39	2	17	19	2	26	28
Tarsiger cyanurus	0	17	17	0	5	5	0	5	5
Emberiza variabilis	4	10	14	1	4	5	4	9	13
Luscinia calliope	6	2	8	3	1	4	6	2	8
Sitta europaea	3	0	3	1	0	1	1	0	1
Troglodytes troglodytes	0	3	3	0	2	2	0	4	4
Erithacus akahige	0	1	1	0	1	1	0	3	3
Phylloscopus schwarzi	1	0	1	1	0	1	2	0	2
Turdus obscurus	1	0	1	1	0	1	1	0	1
Total	812	1,246	2,058	153	551	704	217	1,042	1,259

*Only live ticks on migratory birds were counted.

Appendix Table 2. Information on migratory birds captured in Japan, 2021, and the infesting ticks

Species of migratory bird	No. captured birds			No. birds infested by ticks*			No. ticks*		
	Hamatonbetsu (Lake Kutcharo)	Nemuro (Lake Furen)	Total	Hamatonbetsu (Lake Kutcharo)	Nemuro (Lake Furen)	Total	Hamatonbetsu (Lake Kutcharo)	Nemuro (Lake Furen)	Total
Emberiza spodocephala	651	1,098	1,749	157	421	578	273	896	1,169
Uragus sibiricus	0	61	61	0	1	1	0	1	1
Horornis diphone	51	8	59	9	1	10	9	1	10
Turdus chrysolaus	8	24	32	4	20	24	7	37	44
Emberiza variabilis	7	9	16	4	4	8	13	17	30
Tarsiger cyanurus	0	8	8	0	4	4	0	6	6
Certhia familiaris	2	4	6	1	1	2	1	1	2
Parus minor	6	0	6	1	0	1	1	0	1
Troglodytes troglodytes	0	6	6	0	4	4	0	7	7
Parus palustris	0	5	5	0	1	1	0	1	1
Luscinia calliope	4	0	4	1	0	1	2	0	2
Sitta europaea	0	2	2	0	1	1	0	1	1
Turdus obscurus	1	0	1	1	0	1	1	0	1
Total	730	1,225	1955	178	458	636	307	968	1,275

*Only live ticks on migratory birds were counted.

Appendix Table 3. Information on ticks from which Yezo virus was detected in a study of ticks collected from migratory birds, Japan, 2020–2021

Strain	Tick species	Stage	No. ticks in pool	Status of bloodfeeding		
				Engorged	Unfed	Engorged or unfed
YEZV/tick/BT-1821/Japan/2020	*Ixodes persulcatus*	Nymph	5	1	4	0
YEZV/tick/BT-1822/Japan/2020	*Ixodes persulcatus*	Nymph	5	1	4	0
YEZV/tick/BT-1826/Japan/2020	*Ixodes persulcatus*	Nymph	5	0	5	0
YEZV/tick/BT-1844/Japan/2020	*Ixodes persulcatus*	Larva	10	3	6	1
YEZV/tick/BT-1864/Japan/2020	*Ixodes persulcatus*	Larva	10	0	10	0
YEZV/tick/BT-1968/Japan/2020	*Ixodes persulcatus*	Larva	10	3	1	6
YEZV/tick/BT-2135/Japan/2021	*Ixodes persulcatus*	Nymph	5	0	5	0
YEZV/tick/BT-2155/Japan/2021	*Ixodes persulcatus*	Nymph	5	0	5	0

Appendix Table 4. Pairwise comparison of the nucleotide and amino acid sequences of the RNA-dependent RNA polymerase of Yezo virus strains and Sulina virus IxriSL16-01*

Virus	YEZV/tick/BT-1821/Japan/2020	YEZV/tick/BT-1826/Japan/2020	YEZV/tick/BT-1844/Japan/2020	YEZV/tick/BT-1864/Japan/2020	YEZV/tick/BT-1968/Japan/2020	YEZV/tick/BT-2135/Japan/2021	YEZV/tick/BT-2155/Japan/2021	YEZV/human/HH001-2019/Japan/2019	YEZV/human/HH003-2020/Japan/2020	YEZV/human/HH007-2016 /Japan/2016	YEZV/human/HH008-2017/Japan/2017	YEZV/human/HH009-2017/Japan/2017	YEZV/human/HH011-2020/Japan/2020	YEZV/human/H-IM01/China/2018	YEZV/tick/T-HLJ01/China/2021	YEZV/tick/T-HLJ02/China/2021	YEZV/tick/T-HLJ03/China/2021	YEZV/tick/TIGMIC_1/China/2019	YEZV/tick/TIGMIC_2/China/2019	YEZV/tick/TIGMIC_3/China/2019	YEZV/tick/T-IM01/China/2021	YEZV/tick/T-JL01/China/2020	Sulina virus IxriSL16-01
YEZV/tick/BT-1821/Japan/2020	—	99.8	99.6	98.8	99.1	99.1	99.6	97.3	99.1	97.4	98.8	97.2	97.4	98.4	98.7	98.4	98.8	98.7	98.4	98.5	98.4	98.8	72.8
YEZV/tick/BT-1826/Japan/2020	99.9	—	99.6	98.8	99.1	99.1	99.6	97.2	99.1	97.4	98.8	97.2	97.4	98.3	98.6	98.4	98.8	98.7	98.4	98.5	98.3	98.7	72.8
YEZV/tick/BT-1844/Japan/2020	99.9	99.9	—	98.8	99.1	99.1	99.9	97.2	99.1	97.4	98.8	97.2	97.4	98.3	98.6	98.4	98.8	98.7	98.4	98.5	98.3	98.7	72.8
YEZV/tick/BT-1864/Japan/2020	99.4	99.4	99.5	—	98.9	98.9	98.8	97.3	98.9	97.3	99.7	97.2	97.3	98.1	98.6	98.3	98.7	98.6	98.3	98.4	98.1	98.7	72.8
YEZV/tick/BT-1968/Japan/2020	99.8	99.8	99.9	99.6	—	99.7	99.2	97.5	99.5	97.6	99.0	97.4	97.6	98.4	98.8	98.5	98.9	98.9	98.6	98.6	98.4	98.9	72.8
YEZV/tick/BT-2135/Japan/2021	99.7	99.7	99.8	99.6	99.9	—	99.1	97.4	99.4	97.5	98.9	97.4	97.5	98.4	98.7	98.5	98.9	98.8	98.5	98.6	98.3	98.9	72.8
YEZV/tick/BT-2155/Japan/2021	99.9	99.9	100.0	99.5	99.8	99.8	—	97.3	99.1	97.4	98.8	97.2	97.4	98.4	98.6	98.4	98.8	98.8	98.5	98.5	98.3	98.8	72.8
YEZV/human/HH001-2019/Japan/2019	99.5	99.5	99.6	99.5	99.8	99.7	99.6	—	97.4	98.4	97.2	98.3	98.4	97.4	97.2	97.4	97.4	97.2	97.4	97.5	97.3	97.3	72.9
YEZV/human/HH003-2020/Japan/2020	99.7	99.7	99.8	99.6	99.9	99.9	99.8	99.7	—	97.5	98.9	97.4	97.5	98.4	98.7	98.5	98.9	98.8	98.5	98.6	98.3	98.9	72.8
YEZV/human/HH007-2016 /Japan/2016	99.5	99.5	99.6	99.4	99.7	99.7	99.6	99.8	99.6	—	97.3	99.2	100.0	97.5	97.3	97.5	97.5	97.4	97.6	97.6	97.4	97.4	72.9
YEZV/human/HH008-2017/Japan/2017	99.5	99.5	99.6	99.9	99.8	99.7	99.6	99.6	99.7	99.5	—	97.2	97.3	98.2	98.6	98.3	98.7	98.7	98.3	98.4	98.1	98.7	72.8
YEZV/human/HH009-2017/Japan/2017	99.4	99.4	99.5	99.3	99.6	99.5	99.4	99.7	99.5	99.7	99.4	—	99.2	97.3	97.2	97.4	97.4	97.2	97.4	97.4	97.3	97.3	72.8
YEZV/human/HH011-2020/Japan/2020	99.5	99.5	99.6	99.4	99.7	99.7	99.6	99.8	99.6	100.0	99.5	99.7	—	97.5	97.3	97.5	97.5	97.4	97.6	97.6	97.4	97.4	72.9
YEZV/human/H-IM01/China/2018	99.5	99.5	99.6	99.5	99.7	99.7	99.6	99.7	99.6	99.6	99.6	99.5	99.6	—	98.2	98.5	98.4	98.3	98.5	98.6	100.0	98.3	72.4
YEZV/tick/T-HLJ01/China/2021	99.6	99.6	99.8	99.5	99.9	99.8	99.7	99.7	99.8	99.6	99.6	99.5	99.6	99.7	—	98.5	98.9	98.8	98.4	98.4	98.2	98.8	72.6
YEZV/tick/T-HLJ02/China/2021	99.6	99.6	99.7	99.5	99.8	99.8	99.7	99.8	99.7	99.7	99.6	99.6	99.7	99.7	99.8	—	98.4	98.4	98.6	98.7	98.5	98.3	72.5
YEZV/tick/T-HLJ03/China/2021	99.6	99.6	99.7	99.5	99.8	99.8	99.6	99.6	99.7	99.6	99.6	99.4	99.6	99.6	99.8	99.7	—	99.0	98.5	98.5	98.3	99.0	72.6
YEZV/tick/TIGMIC_1/China/2019	99.7	99.7	99.8	99.5	99.8	99.8	99.8	99.7	99.8	99.7	99.6	99.5	99.7	99.7	99.8	99.8	99.8	—	98.4	98.5	98.2	98.9	72.8
YEZV/tick/TIGMIC_2/China/2019	99.6	99.6	99.7	99.5	99.8	99.8	99.7	99.8	99.8	99.7	99.6	99.6	99.7	99.8	99.8	99.8	99.7	99.8	—	99.0	98.5	98.4	72.9
YEZV/tick/TIGMIC_3/China/2019	99.7	99.7	99.8	99.6	99.9	99.9	99.8	99.8	99.8	99.8	99.7	99.6	99.8	99.8	99.8	99.9	99.8	99.9	100.0	—	98.5	98.5	72.9
YEZV/tick/T-IM01/China/2021	99.5	99.5	99.6	99.5	99.7	99.7	99.6	99.7	99.6	99.6	99.6	99.5	99.6	100.0	99.7	99.7	99.6	99.7	99.8	99.8	—	98.3	72.4
YEZV/tick/T-JL01 /China/2020	99.5	99.5	99.6	99.4	99.8	99.7	99.6	99.6	99.7	99.5	99.5	99.4	99.5	99.6	99.8	99.6	99.8	99.7	99.7	99.7	99.6	—	72.8
Sulina virus IxriSL16-01	82.1	82.2	82.2	82.0	82.1	82.1	82.1	82.2	82.1	82.2	82.1	82.1	82.2	82.0	82.2	82.1	82.1	82.1	82.1	82.1	82.0	82.1	—

*Nucleotide sequence identity (%) is shown above the diagonal and amino acid sequence identity (%) is shown below the diagonal.

Appendix Table 5. Pairwise comparison of the nucleotide and amino acid sequences of the glycoprotein precursor of Yezo virus strains and Sulina virus IxriSL16-01*

Virus	YEZV/tick/BT-1821/Japan/2020	YEZV/tick/BT-1826/Japan/2020	YEZV/tick/BT-1844/Japan/2020	YEZV/tick/BT-1864/Japan/2020	YEZV/tick/BT-1968/Japan/2020	YEZV/tick/BT-2135/Japan/2021	YEZV/tick/BT-2155/Japan/2021	YEZV/human/HH001-2019/Japan/2019	YEZV/human/HH003-2020/Japan/2020	YEZV/human/HH007-2016/Japan/2016	YEZV/human/HH008-2017/Japan/2017	YEZV/human/HH009-2017/Japan/2017	YEZV/human/HH011-2020/Japan/2020	YEZV/human/H-IM01/China/2018	YEZV/tick/T-HLJ01/China/2021	YEZV/tick/T-HLJ02/China/2021	YEZV/tick/T-HLJ03/China/2021	YEZV/tick/T-IM01/China/2021	YEZV/tick/T-JL01/China/2020	Sulina virus IxriSL16-01
YEZV/tick/BT-1821/Japan/2020	—	99.8	99.6	99.3	99.3	97.5	99.6	97.8	99.1	97.5	99.2	97.1	97.5	98.3	98.6	98.5	98.6	98.3	98.5	68.7
YEZV/tick/BT-1826/Japan/2020	99.8	—	99.7	99.4	99.4	97.5	99.7	97.8	99.2	97.5	99.3	97.1	97.5	98.3	98.7	98.6	98.7	98.3	98.6	68.8
YEZV/tick/BT-1844/Japan/2020	99.6	99.8	—	99.4	99.5	97.5	100.0	97.8	99.2	97.5	99.3	97.2	97.5	98.3	98.7	98.6	98.7	98.3	98.7	68.9
YEZV/tick/BT-1864/Japan/2020	99.6	99.9	99.6	—	99.3	97.6	99.4	98.0	99.3	97.6	99.8	97.3	97.6	98.5	98.9	98.7	98.8	98.5	98.8	69.1
YEZV/tick/BT-1968/Japan/2020	99.5	99.7	99.6	99.6	—	97.5	99.5	97.9	99.2	97.5	99.3	97.2	97.5	98.3	98.7	98.5	98.6	98.3	98.6	69.6
YEZV/tick/BT-2135/Japan/2021	99.2	99.4	99.3	99.3	99.2	—	97.5	99.0	97.5	98.3	97.6	98.0	98.3	97.5	97.5	97.7	97.4	97.5	97.4	69.0
YEZV/tick/BT-2155/Japan/2021	99.6	99.9	99.9	99.7	99.7	99.3	—	97.8	99.2	97.5	99.3	97.2	97.5	98.3	98.7	98.6	98.7	98.3	98.7	68.9
YEZV/human/HH001-2019/Japan/2019	99.4	99.6	99.5	99.5	99.4	99.5	99.6	—	97.9	98.7	97.9	98.3	98.7	97.8	97.8	98.0	97.7	97.8	97.7	69.7
YEZV/human/HH003-2020/Japan/2020	99.6	99.7	99.6	99.6	99.5	99.3	99.6	99.5	—	97.4	99.2	97.1	97.4	98.2	98.7	98.5	98.6	98.2	98.6	69.1
YEZV/human/HH007-2016 /Japan/2016	99.4	99.6	99.5	99.5	99.4	99.5	99.6	99.7	99.5	—	97.5	98.8	100.0	97.4	97.4	97.6	97.3	97.4	97.4	69.8
YEZV/human/HH008-2017/Japan/2017	99.5	99.7	99.6	99.9	99.5	99.3	99.6	99.5	99.6	99.5	—	97.2	97.5	98.5	98.9	98.6	98.7	98.5	98.7	69.1
YEZV/human/HH009-2017/Japan/2017	99.3	99.6	99.4	99.4	99.3	99.4	99.5	99.6	99.4	99.6	99.4	—	98.8	97.2	97.2	97.4	97.0	97.2	97.1	69.7
YEZV/human/HH011-2020/Japan/2020	99.4	99.6	99.5	99.5	99.4	99.5	99.6	99.7	99.5	100.0	99.5	99.6	—	97.4	97.4	97.6	97.3	97.4	97.4	69.8
YEZV/human/H-IM01/China/2018	99.4	99.6	99.5	99.5	99.4	99.3	99.6	99.6	99.5	99.6	99.5	99.5	99.6	—	98.3	98.6	98.2	100.0	98.2	69.2
YEZV/tick/T-HLJ01/China/2021	99.5	99.7	99.6	99.6	99.5	99.3	99.6	99.5	99.6	99.5	99.6	99.4	99.5	99.5	—	98.5	98.8	98.3	98.9	68.5
YEZV/tick/T-HLJ02/China/2021	99.6	99.8	99.6	99.6	99.6	99.5	99.7	99.7	99.6	99.7	99.6	99.6	99.7	99.7	99.6	—	98.3	98.6	98.4	69.1
YEZV/tick/T-HLJ03/China/2021	99.6	99.8	99.6	99.6	99.6	99.3	99.7	99.6	99.6	99.6	99.6	99.5	99.6	99.6	99.6	99.7	—	98.2	98.8	69.3
YEZV/tick/T-IM01/China/2021	99.4	99.6	99.5	99.5	99.4	99.3	99.6	99.6	99.5	99.6	99.5	99.5	99.6	100.0	99.5	99.7	99.6	—	98.2	69.2
YEZV/tick/T-JL01 /China/2020	99.5	99.7	99.6	99.6	99.5	99.3	99.6	99.5	99.6	99.5	99.6	99.4	99.5	99.6	99.6	99.6	99.6	99.6	—	69.1
Sulina virus IxriSL16-01	57.0	57.1	57.1	57.0	57.2	57.0	57.1	57.1	57.1	57.1	57.0	57.3	57.1	57.3	57.1	57.1	57.0	57.3	57.1	—

*Nucleotide sequence identity (%) is shown above the diagonal and amino acid sequence identity (%) is shown below the diagonal.

Appendix Table 6. Pairwise comparison of the nucleotide and amino acid sequences of the nucleoprotein of Yezo virus strains and Sulina virus IxriSL16-01*

Virus	YEZV/tick/BT-1821/Japan/2020	YEZV/tick/BT-1826/Japan/2020	YEZV/tick/BT-1844/Japan/2020	YEZV/tick/BT-1864/Japan/2020	YEZV/tick/BT-1968/Japan/2020	YEZV/tick/BT-2135/Japan/2021	YEZV/tick/BT-2155/Japan/2021	YEZV/human/HH001-2019/Japan/2019	YEZV/human/HH003-2020/Japan/2020	YEZV/human/HH007-2016 /Japan/2016	YEZV/human/HH008-2017/Japan/2017	YEZV/human/HH009-2017/Japan/2017	YEZV/human/HH011-2020/Japan/2020	YEZV/human/H-IM01/China/2018	YEZV/tick/T-HLJ01/China/2021	YEZV/tick/T-HLJ02/China/2021	YEZV/tick/T-HLJ03/China/2021	YEZV/tick/T-IM01/China/2021	YEZV/tick/T-JL01 /China/2020	Sulina virus IxriSL16-01
YEZV/tick/BT-1821/Japan/2020	—	99.8	99.3	98.8	99.4	99.4	99.4	93.5	98.9	93.4	98.8	93.1	93.4	98.6	98.6	93.1	98.4	98.6	98.9	75.6
YEZV/tick/BT-1826/Japan/2020	99.8	—	99.5	99.0	99.6	99.6	99.6	93.6	99.1	93.5	99.0	93.2	93.5	98.8	98.8	93.2	98.6	98.8	99.1	75.9
YEZV/tick/BT-1844/Japan/2020	99.8	100.0	—	99.0	99.6	99.6	99.9	93.7	99.2	93.7	99.0	93.3	93.7	98.8	98.8	93.3	98.6	98.8	99.1	66.6
YEZV/tick/BT-1864/Japan/2020	99.8	100.0	100.0	—	99.3	99.3	99.1	93.6	99.1	93.4	99.9	93.2	93.4	98.9	99.0	93.2	98.8	98.9	99.3	66.6

Virus	YEZV/tick/BT-1821/Japan/2020	YEZV/tick/BT-1826/Japan/2020	YEZV/tick/BT-1844/Japan/2020	YEZV/tick/BT-1864/Japan/2020	YEZV/tick/BT-1968/Japan/2020	YEZV/tick/BT-2135/Japan/2021	YEZV/tick/BT-2155/Japan/2021	YEZV/human/HH001-2019/Japan/2019	YEZV/human/HH003-2020/Japan/2020	YEZV/human/HH007-2016/Japan/2016	YEZV/human/HH008-2017/Japan/2017	YEZV/human/HH009-2017/Japan/2017	YEZV/human/HH011-2020/Japan/2020	YEZV/human/H-IM01/China/2018	YEZV/tick/T-HLJ01/China/2021	YEZV/tick/T-HLJ02/China/2021	YEZV/tick/T-HLJ03/China/2021	YEZV/tick/T-IM01/China/2021	YEZV/tick/T-JL01/China/2020	Sulina virus IxriSL16-01
YEZV/tick/BT-1968/Japan/2020	99.8	100.0	100.0	100.0	—	99.9	99.7	93.7	99.3	93.6	99.3	93.3	93.6	99.1	99.1	93.3	98.9	99.1	99.3	66.5
YEZV/tick/BT-2135/Japan/2021	99.8	100.0	100.0	100.0	100.0	—	99.7	93.7	99.3	93.6	99.3	93.3	93.6	99.1	99.1	93.3	98.9	99.1	99.3	66.4
YEZV/tick/BT-2155/Japan/2021	99.8	100.0	100.0	100.0	100.0	100.0	—	93.7	99.3	93.8	99.1	93.3	93.8	98.9	98.9	93.3	98.7	98.9	99.2	66.6
YEZV/human/HH001-2019/Japan/2019	99.6	99.8	99.8	99.8	99.8	99.8	99.8	—	93.7	97.5	93.6	99.2	97.6	94.0	93.6	98.1	93.8	93.8	93.7	76.1
YEZV/human/HH003-2020/Japan/2020	99.8	100.0	100.0	100.0	100.0	100.0	100.0	99.8	—	93.6	99.1	93.3	93.6	98.8	98.9	93.4	98.7	98.8	99.2	66.6
YEZV/human/HH007-2016/Japan/2016	99.4	99.6	99.6	99.6	99.6	99.6	99.6	99.8	99.6	—	93.9	97.2	99.9	94.0	93.6	97.2	93.5	93.9	93.7	76.6
YEZV/human/HH008-2017/Japan/2017	99.6	99.8	99.8	99.8	99.8	99.8	99.8	99.6	99.8	99.4	—	93.2	93.9	98.9	99.0	93.2	98.8	98.9	99.3	66.6
YEZV/human/HH009-2017/Japan/2017	99.4	99.6	99.6	99.6	99.6	99.6	99.6	99.8	99.6	99.6	99.4	—	97.3	93.6	93.2	98.0	93.4	93.4	93.3	76.1
YEZV/human/HH011-2020/Japan/2020	99.6	99.8	99.8	99.8	99.8	99.8	99.8	100.0	99.8	99.8	99.6	99.8	—	94.0	93.6	97.2	93.5	93.9	93.7	76.6
YEZV/human/H-IM01/China/2018	99.6	99.8	99.8	99.8	99.8	99.8	99.8	99.6	99.8	99.4	99.6	99.4	99.6	—	98.8	93.6	98.6	99.9	99.1	66.4
YEZV/tick/T-HLJ01/China/2021	99.6	99.8	99.8	99.8	99.8	99.8	99.8	99.8	99.8	99.6	99.6	99.6	99.8	99.6	—	93.0	98.9	98.8	99.3	75.6
YEZV/tick/T-HLJ02/China/2021	99.6	99.8	99.8	99.8	99.8	99.8	99.8	100.0	99.8	99.8	99.6	99.8	100.0	99.6	99.8	—	93.5	93.4	93.3	75.7
YEZV/tick/T-HLJ03/China/2021	99.4	99.6	99.6	99.6	99.6	99.6	99.6	99.6	99.6	99.4	99.4	99.4	99.6	99.4	99.8	99.6	—	98.6	99.1	66.2
YEZV/tick/T-IM01/China/2021	99.6	99.8	99.8	99.8	99.8	99.8	99.8	99.6	99.8	99.4	99.6	99.4	99.6	100.0	99.6	99.6	99.4	—	99.1	66.5
YEZV/tick/T-JL01/China/2020	99.6	99.8	99.8	99.8	99.8	99.8	99.8	99.8	99.8	99.6	99.6	99.6	99.8	99.6	100.0	99.8	99.8	99.6	—	66.4
Sulina virus IxriSL16-01	60.5	60.5	60.5	60.5	60.5	60.5	60.5	60.5	60.5	60.3	60.5	60.3	60.5	60.5	60.5	60.5	60.7	60.5	60.5	—

*Nucleotide sequence identity (%) is shown above the diagonal and amino acid sequence identity (%) is shown below the diagonal.
